# Cannabinoids induce cell death in leukaemic cells through Parthanatos and PARP-related metabolic disruptions

**DOI:** 10.1038/s41416-024-02618-6

**Published:** 2024-03-09

**Authors:** M. Medrano, M. Contreras, T. Caballero-Velázquez, L. Martínez, J. A. Bejarano-García, R. Calderón-Ruiz, C. B. García-Calderón, I. V. Rosado, J. A. Pérez-Simón

**Affiliations:** 1 Instituto de Biomedicina de Sevilla (IBIS/CSIC), Universidad de Sevilla, Seville, Spain; 2 Department of Hematology, University Hospital Virgen del Rocío, Universidad de Sevilla, Seville, Spain; 3 Department of Medical Biochemistry, Molecular Biology and Immunology, Universidad de Sevilla, Seville, Spain; 4 Centro Andaluz de Biología Molecular y Medicina Regenerativa (CABIMER), Universidad de Sevilla-CSIC-Universidad Pablo de Olavide, Seville, Spain

**Keywords:** Drug development, Acute myeloid leukaemia

## Abstract

**Background:**

Several studies have described a potential anti-tumour effect of cannabinoids (CNB). CNB receptor 2 (CB2) is mostly present in hematopoietic stem cells (HSC). The present study evaluates the anti-leukaemic effect of CNB.

**Methods:**

Cell lines and primary cells from acute myeloid leukaemia (AML) patients were used and the effect of the CNB derivative WIN-55 was evaluated in vitro, ex vivo and in vivo.

**Results:**

We demonstrate a potent antileukemic effect of WIN-55 which is abolished with CB antagonists. WIN-treated mice, xenografted with AML cells, had better survival as compared to vehicle or cytarabine. DNA damage-related genes were affected upon exposure to WIN. Co-incubation with the PARP inhibitor Olaparib prevented WIN-induced cell death, suggesting PARP-mediated apoptosis which was further confirmed with the translocation of AIF to the nucleus observed in WIN-treated cells. Nicotinamide prevented WIN-related apoptosis, indicating NAD+ depletion. Finally, WIN altered glycolytic enzymes levels as well as the activity of G6PDH. These effects are reversed through PARP1 inhibition.

**Conclusions:**

WIN-55 exerts an antileukemic effect through Parthanatos, leading to translocation of AIF to the nucleus and depletion of NAD+, which are reversed through PARP1 inhibition. It also induces metabolic disruptions. These effects are not observed in normal HSC.

## Introduction

Acute myeloid leukaemia (AML) is characterised by the proliferation of immature clonal myeloid cells [1]. Although the identification of novel targets has allowed to individualise treatment strategies based on specific molecular patterns, the outcome of AML patients remains unsatisfactory [2]. Therefore, new chemotherapeutics approaches are needed.

Therapeutic interest on cannabinoids (CNB) emerged after the discovery of the endocannabinoid system (eCS) [3, 4]. Mammalian tissues express at least two CNB receptor types, CB1 [5] (CNR1), predominantly expressed in the central nervous system, and CB2 [6] (CNR2), mostly present in hematopoietic cells [7].

One of the most relevant therapeutic applications of CNB is its potential anti-tumoral properties [8–10]. In this regard, our group has previously described the antimyeloma effect of CNB [11]. CNB induce apoptosis in different cancer cells through the cleavage of PARP and caspases [12–14], overproduction of ROS [13], inhibition of ERK and AKT signalling [15] or accumulation of ceramides and endoplasmic reticulum (ER) stress [16]. Also, CNB promote increase mitochondrial membrane permeability [17].

Furthermore, the cannabinoid system contributes to the control of metabolism [8]. Accordingly, CB2 restricts the glucose and energy supply of B-lymphoid cells lineage [18]. Moreover, it has been described that CNB inhibit cellular respiration in brain [19] and human oral cancer cells [20] and energetic metabolism in pancreatic cancer cells [21].

The present study was undertaken to evaluate the potential anti-leukaemic effect of CNB and investigate the different pathways and metabolic alterations involved in the trigger of apoptosis.

## Methods

### Statement of ethics

All research involving human material or animal samples were approved by the Ethical Committee for Clinical Research (CEIC) of the University Hospital Virgen del Rocío and was conducted in accordance with the Declaration of Helsinki.

### Cell cultures, drugs and treatments

HL60, MV-4-11 and KG-1a human AML cell lines (ATCC) were cultured in IMDM medium, and U937 cell line (DMSZ, Braunschweig, Germany) was cultured in complete RPMI 1640. All growth media were supplemented with 100 units/mL penicillin, 100 µg /mL streptomycin and 10% foetal bovine serum (FBS). All cells were cultured in a humidified atmosphere of CO_2_/air (5%/95%) and 37 °C. All experiments were performed at least in triplicate and HL60 and U937 cell lines, the prior being shown in main figures and the latter in supplementary figures, except for the metabolic assays in which the data obtained with U937 cells are shown in the main figures because it displays a more active glycolytic pattern. Ex vivo cell selection and drug description is specified in Supplementary material and methods section.

### Cell viability and apoptosis assays

Cell lines were cultured (5 × 10^5^ cells/well) at the indicated concentrations of WIN-55 or DMSO in triplicate wells for 18, 48 and 72 h. Isolated primary cells were cultured (5 × 10^5^ cells/well) for 18 h with DMSO (control) or WIN-55 with or without CB antagonists. WIN-55 was added at different concentrations and time-points. Cell viability was determined by Cell Counting Kit (CCK-8) assay as per manufacturer’s instructions (Dojindo Molecular Technologies). Optical densities were measured at 450 nm using a plate reader MultiskanTM Go Microplate (Thermo Fisher Scientific, Waltham, MA, USA).

AML cells from patients’ BM were identified at diagnosis with a combination of monoclonal antibodies against AML cells-associated antigens at diagnosis (anti-CD33, anti-CD34, anti-CD117, and anti-CD45 [BD Biosciences]). Apoptosis was assessed by Annexin V/7AAD staining assay kit, as per manufacturer’s instructions (BD Biosciences) and analysed on a FACS Canto II Flow Cytometer (BD Biosciences) and analysed with Infinicyt^TM^ Software (Cytognos, Spain).

Other studies, such as oxygen consumption rate (OCR) and extracellular acidification rate (ECAR), western blot, mitochondrial damage or enzyme activity assays are detailed in the supplementary files.

### AML murine model

NOD/scid/IL-2R gamma chain null (NSG) mice were purchased from Charles River Laboratories International (L’Arbresle, France) and maintained with food and water ad libitum, under specific pathogen-free conditions. 8- to 12-week-old NSG mice, were intravenously inoculated with 5 × 10^6^ HL60 or Luc^+^ HL60 cells. Upon confirmation of leukaemic BM engraftment, WIN-55 (5 mg/kg/day) or cytarabine (ARA-C, 50 mg/kg) was administered intraperitoneally and disease progression was monitored overtime by scoring weight loss, percentage of infiltrated human CD45^+^ cells in BM by flow cytometry or bioluminescence assays. Spleen size and human CD45^+^ cell infiltration in peripheral blood (PB), spleen and bone marrow (BM) were analysed 5 days post-treatment.

The effect of cannabinoids on normal hematopoiesis was evaluated on healthy BALB/c mice. WIN-55 (5 mg/kg/day) was intraperitoneally administered daily for 7 and 28 days respectively. BM and PB cell populations were analysed by flow cytometry and blood count assays. The antibodies used are listed in Supplementary Table 2.

## Results

### Human AML cell lines but not healthy primary cells are sensitive to the cytotoxic effects of WIN-55

**Fig. 1:**
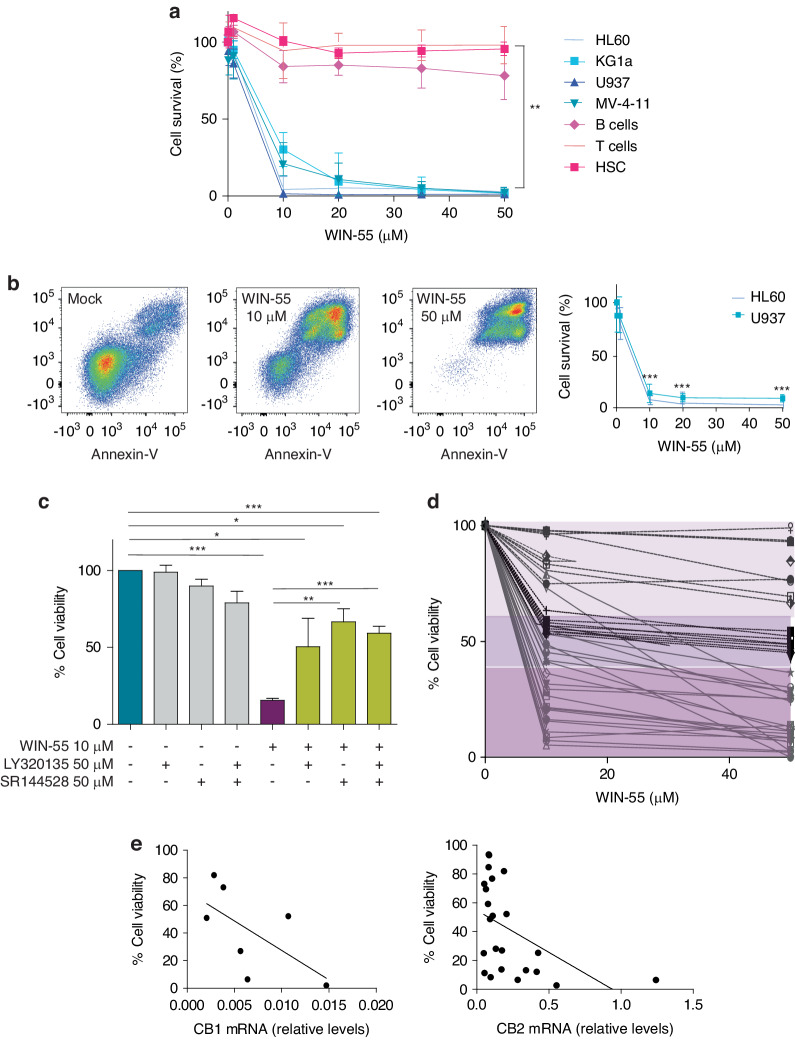
Human AML cell lines are sensitive to the cytotoxic effects of WIN-55 in vitro and CB2 receptor is involved in the process. **a** Effect of WIN-55 on AML cell lines and healthy primary cells. Cell viability and/or proliferation were assessed for the AML cell lines HL60, KG-1a, U937 and MV-4-11 and three populations of normal cells (HSC, T lymphocytes and B lymphocytes), previously isolated by AutoMACS, after 18 h of incubation with WIN-55 at different concentrations by using the Cell Counting Kit (CCK-8) assay. **b** Cell viability analysis of HL60 and U937 cell lines after 18 h incubation with vehicle or WIN-55 (10 µM or 50 µM doses) was assessed by 7AAD/Annexin V labelling using FACS analysis. **c** Effect of the CB1 (LY320135) and/or CB2 (SR144528) antagonists in HL60 cells viability assessed after 18 h of incubation with LY320135 or SR144528 alone or in combination with 10 µM WIN55 by using the CCK-8 assay. The data are mean ± SD for n = 4 replicates. Statistically significant differences were determined by Student’s *t* tests: *p < 0.05, **p ≤ 0.005 and ***p ≤ 0.0005. **d** Effect of WIN-55 ex vivo. Mononuclear cells from 40 AML patients were incubated for 18 h with 10 µM and 50 µM WIN-55 and cell viability was analysed by 7-AAD/Annexin V labelling using FACS analysis. The three different tones of colours mean three groups according to the WIN-55 sensitivity. **e** Correlation between the antileukemic effect of WIN-55 after 18 h incubation at 50 µM dose and the expression levels of CB1 and CB2 receptors. Cells from patient samples with BM infiltration >75% blasts were used either to assess the cell viability by 7AAD/Annexin V labelling using FACS analysis or to measure the expression levels of CB1 and CB2 receptors by quantitative PCR. The data are mean ± SD for n = 6 and n = 23 replicates respectively. Statistically correlations were determined by Spearman r analysis.

We examined the cytotoxic effect of WIN-55 on tumour cell viability in vitro. To this end, AML cell lines (U937, HL60, KG-1a, and MV-4-11) and healthy primary cells (isolated HSC, B, and T cells) were exposed to various concentrations of WIN-55 for 18 h and cell viability was measured by Cell Counting Kit (Fig. 1a). Exposure to ≥10 µM WIN-55 led to a significant reduction in the viability of AML cell lines in a concentration-dependent manner. On the contrary, the viability of normal B, T and CD34+ cell subpopulations from healthy donors remained unaffected. The antiproliferative effect of WIN-55 was concentration- and time-dependent (Supplementary Fig. 1A).

Annexin V and 7-AAD staining by flow cytometry also showed that WIN-55 increases apoptosis (Fig. 1b). In addition, we also observed that a short treatment of only 15 min of WIN-55 is enough to activate the apoptosis observed later. (Supplementary Fig. 1B).

We next exposed HL60 cells to WIN-55 (10 µM) in the presence or absence of CB1 (LY320135) or/and CB2 (SR144528)-antagonists and cell viability was determined 18 h later. The results showed that treatment with CB2-selective antagonists significantly inhibit the cannabinoid-induced cell death (Fig. 1c).

### WIN-55 exerts a proapoptotic effect in primary cells from AML patients

The effect of WIN-55 (50 µM) was further examined in AML cells obtained from 40 patients at diagnosis (Supplementary Table 1 and Supplementary Fig. 1C). Remarkably, AML blasts viability was significantly affected (>45% drop) from a large proportion of patients (77.5%) (Fig. 1d).

We next investigated whether the effect of WIN-55 in primary AML cells was related to the expression levels of cannabinoid receptors. We observed an inverse correlation between receptor expression and viability after treatment (Fig. 1e). Specifically, the CB2 receptor showed a significant inverse correlation (*p* = 0.0134) whereas the CB1 receptor showed a clear but not significant trend.

### WIN-55 cannabinoid produces a potent and selective antileukemic in vivo effect

**Fig. 2:**
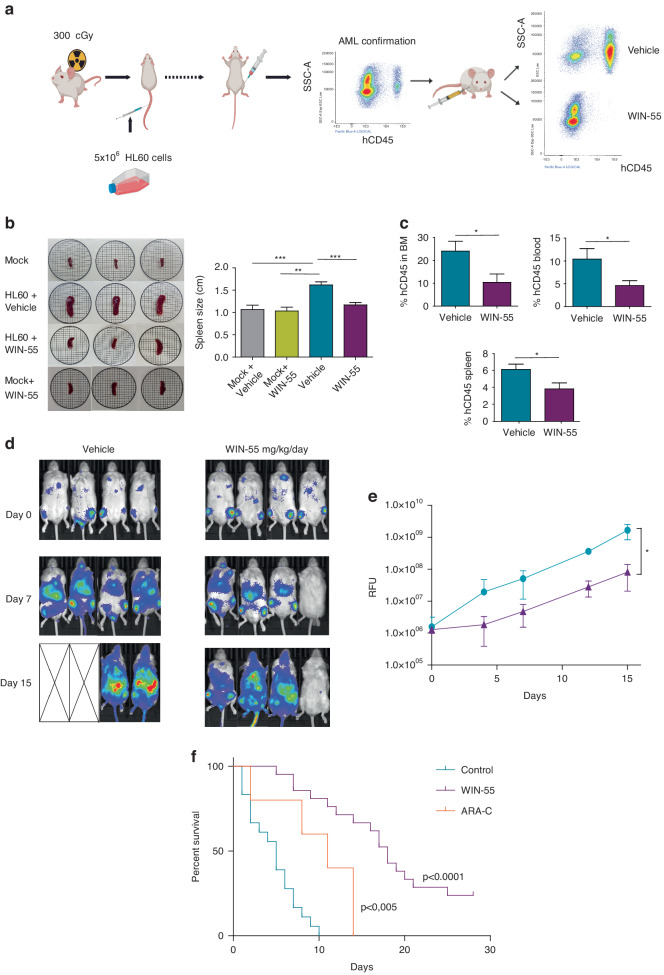
Effect of WIN-55 in vivo. **a** Experimental procedure carried out with the AML in vivo model to generate xenografts with the HL60 and HL60-Lucif cell lines. **b** Representative image of the spleens mean size for each group after 5 days of treatment (left) and the average quantification of the measurements taken from all the mice expressed in cm (right). The data are mean ± SD for n = 7 replicates. Statistically significant differences were determined by Student’s *t* tests: **p ≤ 0.005 and ***p ≤ 0.0005. **c** FACS analysis for human CD45^+^ cells in BM, PB and spleen. Mice were xenotransplanted with HL60 cells and were treated during 5 days with vehicle or WIN-55 at a dose of 5 mg/kg/day. The data are mean ± SD for n = 7 replicates. Statistically significant differences were determined by Student’s *t* tests: *p < 0.05. **d** Murine model xenografted with HL60-Luciferase cells. Infiltration was measured by bioluminescence after luciferin injection. Mice were arbitrarily assigned to 2 groups of 7 mice: vehicle-treated or 5 mg/kg/day WIN-55 treated once the BM infiltration was confirmed. **e** The bioluminescence intensity of mice treated with vehicle (blue line) versus those treated with WIN-55 (purple line). The bioluminescence intensity was measured and expressed in relative fluorescence units (RFU). The data are mean ± SD for n = 7. **f** Survival of WIN55-treated mice compared to the vehicle-treated mice and the ARA-C-treated mice. Treatment was performed for 5 days at 50 mg/kg for ARA-C, 5 mg/kg/day for WIN-55 or vehicle. Data are represented in a Kaplan Meyer plot. Data are provided as mean ± SD for n = 21 for WIN-55-treated group, n = 18 for control group, and n = 10 for the ARA-C-treated group.

Immunocompromised NSG mice were injected intravenously with HL60 cells. Once BM infiltration was confirmed by flow cytometry or bioluminescence, we treated the mice with 5 mg/kg/day WIN-55 or vehicle (Fig. 2a).

The spleen size was measured after 5 days of treatment. We observed an increase of spleen size in HL60 xenograft mice vs. mock mice without AML, which was reversed after WIN-55 treatment (Fig. 2b). In addition, WIN-55 also induced an extensive reduction of HL60 cell numbers in PB, BM, and spleen (Fig. 2c).

AML progression was monitored by bioluminescence assays which showed an intensity reduction in WIN-55-treated vs. vehicle-treated mice (Fig. 2d, e). Moreover, we observed a significant increase in survival among treated mice (Fig. 2f).

To evaluate the toxicity profile of WIN-55 in healthy hematopoiesis, BALB/c mice were treated with 5 mg/kg/day of WIN-55 for 7 and 28 days and the different PB and HSC subpopulations were analysed (Supplementary Fig. 2A).

Peripheral blood population assays revealed a slight and transient decrease in monocytes upon treatment with WIN-55 on day 7, which returned to normal levels after 28 days of treatment. An increase in platelet counts and no significant changes for the rest of blood cell counts were observed upon treatment (Supplementary Fig. 2B).

Furthermore, different HSC subpopulations were analysed by flow cytometry (Supplementary Fig. 2C). We confirmed that WIN-55 did not induce significant changes in Lin^-^Kit^+^Sca-1^+^ (LKS) or common lymphoid progenitors (CLP) subpopulations. An increase of total Lin^-^Kit^+^Sca-1^-^ (LK) cells was observed after 7 days of treatment. This increase was not observed after 28 days of treatment. After 28 days of WIN-55, a slight decrease in the number of total LK cells was observed, which was attributed to megakaryocyte/erythroid progenitors (MEP) subpopulation (Supplementary Fig. 2D).

Finally, to evaluate whether the pro-apoptotic effect of WIN-55 could be related to cell cycle, BALB/c mice were treated with G-CSF with or without WIN-55. The effect of WIN-55 was similar irrespective of the previous treatment with G-CSF (data not shown).

### WIN-55 dysregulates key proliferation pathways and induces caspase-mediated apoptosis in HL60 cells

To elucidate the signalling pathways involved in the antileukemic effect of WIN-55, we performed microarray assays in HL60 cells exposed or not to WIN-55.

Gene Set Enrichment Analysis (GSEA) using Gene Ontology (GO) database revealed statistically significant differences between WIN-55-treated and control samples for genes associated with processes such as response to ER stress (upregulation), metabolic process (downregulation), DNA repair (downregulation), mitochondrion organisation (downregulation), or cell proliferation (downregulation) (Table 1 and Supplementary Fig. 3).

**Table 1:** Disregulated pathways identified upon WIN-55 treatment in HL-60 (AML) cell line.

ID	Description	NES	FDR	Gene Set Size	Nº of upregulated genes	Nº of downregulated genes
GO:0034976	Response to endoplasmic reticulum stress	2.80682166	9.9225E-33	283	42	1
GO:0008152	Metabolic process	-1.326297	1.8239E-26	10815	169	70
GO:0006281	DNA repair	-1.6245726	1.6789E-07	525	4	5
GO:0007005	Mitochondrion organisation	-1.6094804	3.7675E-07	510	9	3
GO:0008283	Cell population proliferation	-1.2979225	3.2014E-06	1837	32	23

**Fig. 3:**
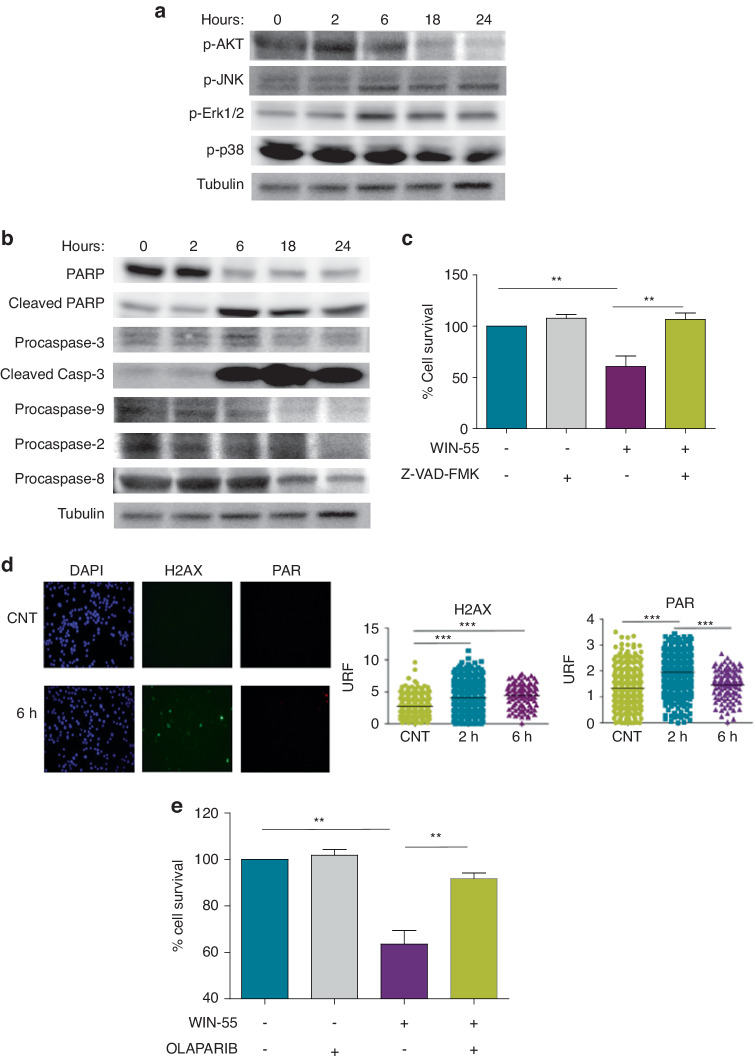
Study of signalling pathways affected by WIN-55. **a** Western blotting analysis of the phosphorylation of total protein fractions (n = 3) related to MAPK and PI3K/Akt signalling pathways. HL60 cells were incubated with 50 µM of WIN-55 for different incubation periods (from 0 to 24 h). **b** Western blotting analysis of total fractions of the cleaved forms of caspase 3, the cleavage of its substrate PARP, and the initiator caspases Casp-9, Casp-2 and Casp-8. HL60 cells were incubated with 50 µM WIN-55 for different incubation periods (from 0 to 24 h). **c** Cell viability was assessed for HL60 after 18 h of incubation with vehicle, 5 µM WIN-55 alone or in combination with 10 µM pan-caspase inhibitor Z-VAD(OMe)-FMK by using the CCK-8 assay. **d** Immunofluorescence images of γ-H2AX and PAR staining in HL60 cells treated with WIN-55 (50 µM) for 6 h (left) and dot plot showing the quantification of nuclear mean fluorescence intensity (right). **e** Cell viability was assessed for HL60 after 18 h of incubation with 10 µM WIN-55 or 10 µM Olaparib alone or in combination by using the CCK-8 assay. The data are mean ± SD for n = 3. Statistically significant differences were determined by Student’s *t* tests: *p < 0.05, **p ≤ 0.005 and ***p ≤ 0.0005.

To validate the transcriptomic data, we analysed the protein expression levels of AKT, ERK, JNK, and p38-MAPK [22, 23]. WB assays demonstrated that WIN-55 slightly upregulated p-JNK and p-Erk1/2 whereas moderately down-regulated p-p38-MAPK and p-AKT over time (Fig. 3a), indicating that WIN-55 down-modulates cell proliferation-inducing pathways while up-modulating stress response proteins.

WIN-55 treatment also led to the activation of caspases (Fig. 3b). More specifically, we observed cleavage of caspase-3 and PARP, and reduction in procaspase-2, -8 and -9. To further investigate the importance of caspases in the cytotoxic effect of WIN-55, cells were pre-incubated with the pan-caspase inhibitor Z-VAD(OMe)-FMK for 60 min and then, WIN-55 was added for 18 h. As shown in Fig. 3c, the pro-apoptotic effect of cannabinoids on AML cells significantly decreased upon co-culture with pan-caspase inhibitors. Therefore, these results provide evidence that WIN-55 induces apoptosis-mediated cell death by activating both the intrinsic and the extrinsic pathways.

### WIN-55 does not cause direct DNA damage, but PARP1 plays an important role in the effect on AML cells

Besides proliferation and apoptosis, we also tested whether WIN-55 treatment produces DNA damage in AML cells. Immunofluorescence images revealed a low increase of γ-H2AX and nuclear PARylation (Fig. 3d) which could be mostly attributed to the presence of apoptotic cells after incubation with WIN-55. To rule out a direct effect on DNA damage, we added Olaparib, Talazoparib and Niraparib (PARP inhibitors). A synergistic effect between both compounds should have been observed in case of a direct DNA damage. By contrast, Olaparib reversed almost 100% of the death caused by WIN-55 (Fig. 3e), and Talazoparib and Niraparib also reversed, in part, the proapoptotic effect produced by WIN.55 (Supplementary Fig. 4A). Accordingly, we confirmed a significant involvement of PARP in the antileukemic effect of WIN-55.

### WIN-55 induces damage to organelles such as the endoplasmic reticulum and mitochondria

**Fig. 4:**
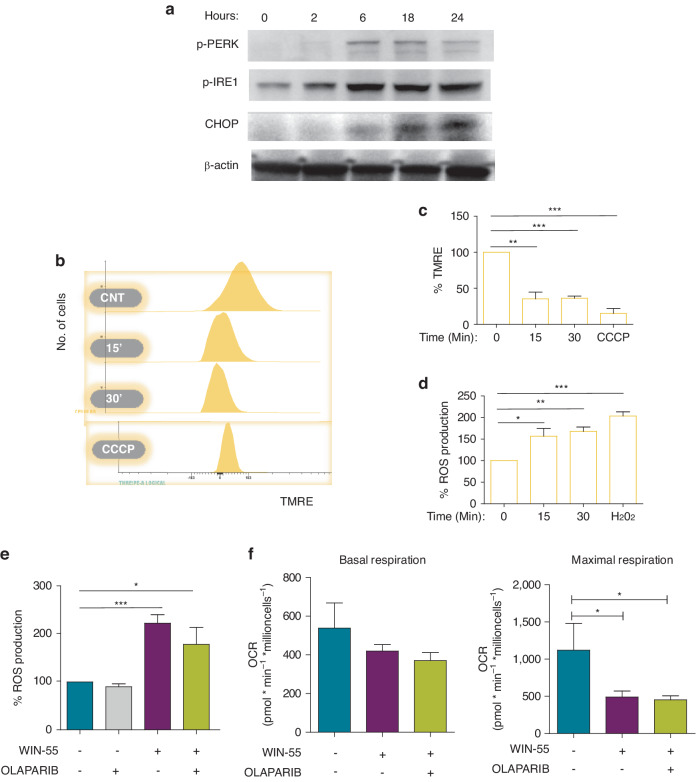
WIN-55 induces damage to endoplasmic reticulum and mitochondria. **a** Western blotting analysis of total protein fractions involved in the UPR. HL60 cells were incubated with 50 µM WIN-55 for the indicated time period. Data are provided as mean ± SD of n = 3. **b** Mitochondrial membrane potential of HL60 cells non-treated cells versus WIN-55 treated cells using FACS analysis. HL60 cells were incubated with 50 µM WIN-55 for the indicated time period. Non-treated and 50 µM WIN-55 treated HL60 cells (10^6^ cells per assay at 15 and 30 min) were stained with tetramethylrhodamine, ethyl ester (TMRE). Carbonyl cyanide m-chlorophenyl hydrazine (CCCP), an inhibitor of mitochondrial oxidative phosphorylation, was used as a positive control for the loss of Δψm. Data are provided as mean ± SD of n = 4. **c** Mitochondrial membrane potential loss of non-treated versus WIN55-treated HL60 cells using Fluoroscan multiwell plate reader. Data are provided as mean ± SD of n = 3. **d** Intracellular reactive oxygen species (ROS) levels of non-treated versus WIN-55 treated cells using MitoSOX assay. HL60 cells were stained with 5 µM of the MitoSOX probe to detect mitochondrial superoxide using a fluorescence plate reader. H_2_O_2_ was used as a positive control for ROS production. Data are provided as mean ± SD of n = 3. **e** Intracellular reactive oxygen species (ROS) levels of HL60 cells non-treated versus WIN55-treated or Olaparib-treated alone or in combination using MitoSOX assay. HL60 cells were treated with 50 µM WIN-55 for 30 min, 10 µM Olaparib preincubated 1 h before WIN55 treatment, or both. Data are provided as mean ± SD of n = 4. **f** Oxygen consumption rate (OCR) values were measured in HL60 cells during sequential injection of oligomycin, CCCP, and Rot+Ama in HL60 cells after WIN-55 and/or Olaparib treatments using a Seahorse Analyzer. Basal and maximal respiration of cells were calculated. Data are provided as mean ± SD of n = 3. Statistically significant differences were determined by Student’s *t* tests: *p < 0.05, **p ≤ 0.005 and ***p ≤ 0.0005.

The fact that transcriptomic data revealed an up and downregulation of ER stress and mitochondrion organisation processes, respectively (Supplementary Fig. 3), lead us to hypothesise that the dysregulation of these processes might drive the antileukemic effect of WIN-55. For this reason, we analysed the activation of ER stress key proteins for the Unfolded Protein Response (UPR). WB assays showed that WIN-55 significantly increased the expression of p-PERK, p-IRE1, and CHOP proteins in AML cells (Fig. 4a).

Furthermore, the effect of WIN-55 on the functional activity of mitochondria was also studied. HL60 cells were exposed to 50 µM WIN-55 for 15 or 30 min. Results showed a significant reduction of the mitochondrial membrane potential (Fig. 4b, c).

Next, we studied the reactive oxygen species (ROS) production by performing a MitoSox assay. Exposure of HL60 cells to WIN-55 led to a significant increase in ROS levels at 15 min or more (Fig. 4d). Interestingly, this effect was not observed in normal CD34^+^ cells (Supplementary Fig. 4B). Moreover, since PARP has been reported to play a protective role in mitochondria by preventing ROS production [24], we measured the ROS levels after the combination treatment of WIN-55 and Olaparib in HL60 cells. Pre-incubation with Olaparib did not prevent the increase of ROS levels caused by WIN-55 treatment (Fig. 4e).

Secondly, mitochondrial respiration of AML versus CD34^+^ cells was studied by determining the OCR. Of note, only basal respiration could be analysed in normal CD34^+^ cells due to cell amount limitation. Regarding AML cell lines, both basal and maximal mitochondrial respiration were decreased upon exposure to WIN-55 and this decrease was not reversed by Olaparib (Fig. 4f and Supplementary Fig. 4C). It is also worth mentioning that in the case of the HL60 basal respiration, we observed a clear downward trend, although the differences were not statistically significant due to a higher scatter of data in the control condition. By contrast, the OCR value of CD34^+^ cells was not altered by WIN-55 treatment (Supplementary Fig. 4D), meaning that the effect of WIN-55 treatment on the mitochondrial respiration is also an exclusive effect on AML cells.

### WIN-55 alters crucial metabolic pathways of AML cells

Since the transcriptomic data exhibited significant differences between WIN-55-treated and control samples for genes associated with metabolic processes, we aimed to evaluate different metabolic pathways that could be altered upon exposure to WIN-55 in AML cells.

**Fig. 5:**
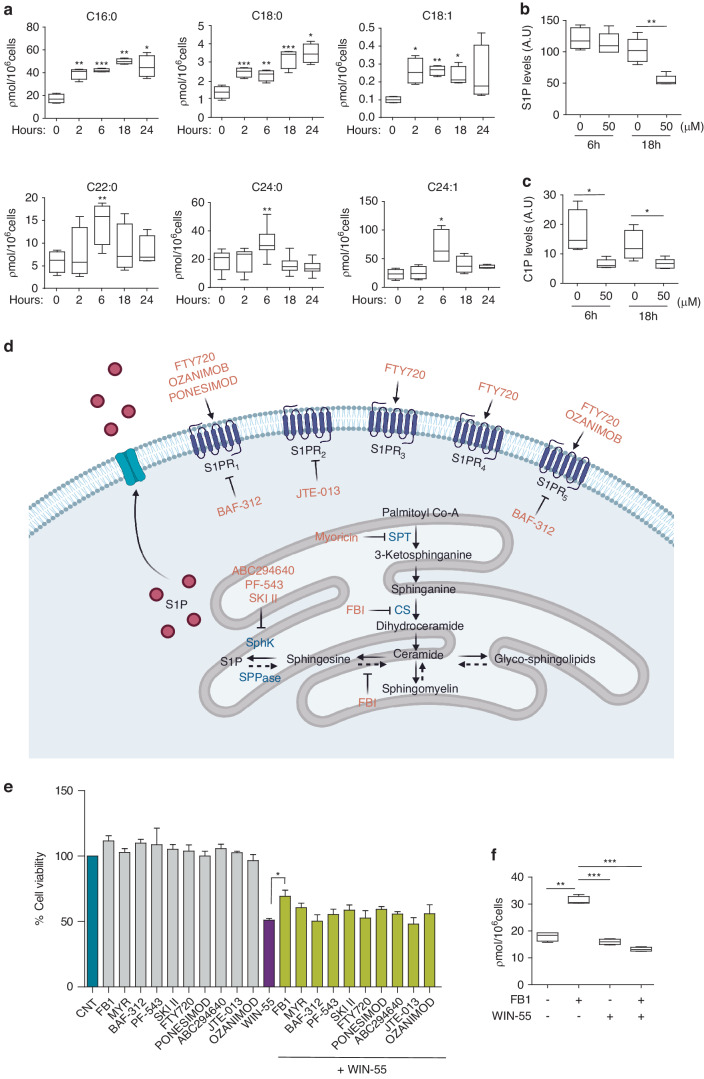
Role of ceramides in the antileukemic effect of WIN-55. **a** Quantification levels of the different types of ceramides in non-treated versus WIN-55-treated HL60 cells by HPLC-MS/MS analysis. HL60 cells were incubated with 50 µM WIN-55 at 2, 6, 18 and 24 h. Data are provided as mean ± SD of n = 3. C16:0, C18:0 and C18:1 ceramides showed a steadily increasing pattern in both AML cell lines up to 24 h, while longer chain ceramides peaked at 6 h post-exposure and then returned to basal levels. **b** Quantification levels of S1P in non-treated versus WIN-55-treated HL60 cells by HPLC-MS/MS analysis. **c** Quantification levels of C1P in non-treated versus WIN-55-treated HL60 cells by HPLC-MS/MS analysis. Sphingosine-1-phosphate (S1P) and ceramide-1-phosphate (C1P) levels were also altered in HL60 cells after WIN-55 treatment at both 6 and 18 h. For (**a**), the ceramide 17:0 was used as internal standard and relative standard deviations (RSD) were <10%. Data are provided as mean ± SD of n = 3. **d** Scheme of the de novo ceramide synthesis pathway and the inhibitory steps (in red) for the inhibitors used in this study. **e** Cell viability was assessed for HL60 cells after incubation with the here showed ceramide inhibitors alone or in combination with WIN-55 treatment by using the CCK-8 assay. Only FB1 inhibitor partially prevented WIN-55-mediated HL60 cell death. Data are provided as mean ± SD of n = 3. Statistically significant differences were determined by Student’s *t* tests: *p < 0.05. **f** Quantification levels of ceramides in HL60 cells upon Fumonisin B1 treatment by using HPLC/MS-MS analysis. HL60 cells were incubated for 6 h with 50 µM WIN-55 in the presence of Fumonisin B1 or vehicle. The graph shows the levels of ceramide C16:0 as an example. Data are provided as mean ± SD of n = 3. Statistically significant differences were determined by Student’s *t* tests: **p ≤ 0.005 and ***p ≤ 0.0005.

The involvement of de novo synthesised ceramides in cannabinoid-induced apoptosis has been described [22]. Accordingly, we performed HPLC assays and confirmed an increase of long (C16: 0-C20: 1) and very long chain ceramides (C22: 0-C24: 1) after exposure to WIN-55. Remarkably, pre-treatment with different inhibitors of ceramide synthesis only partially prevented WIN-induced apoptosis (Fig. 5 and Supplementary Fig. 5). Consequently, we can affirm that ceramide plays a minor role in the proapoptotic effect of cannabinoids on leukaemic cells, and therefore this is not the main mechanism triggering cell death.

**Fig. 6:**
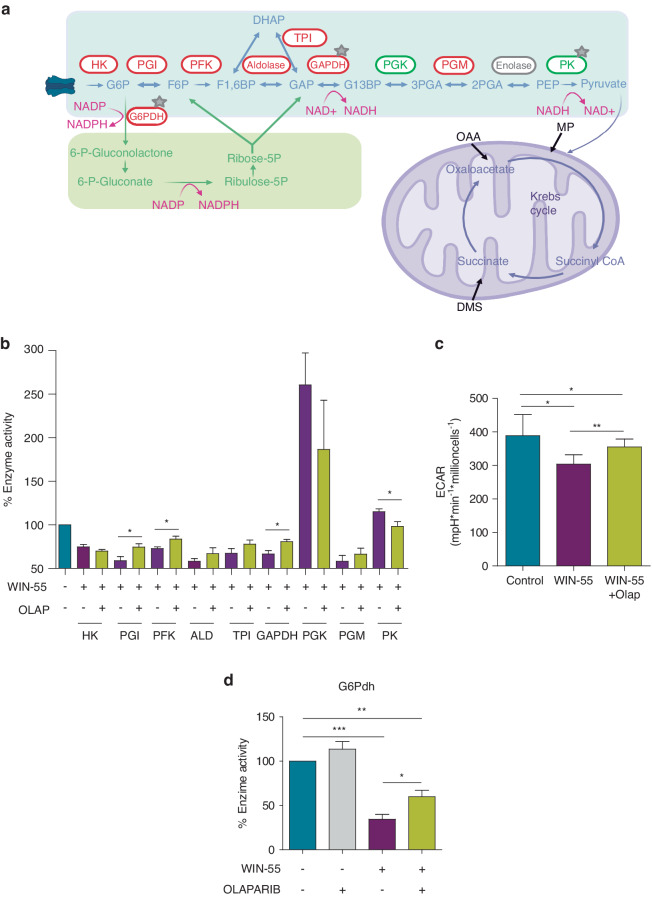
WIN-55 alters crucial metabolic pathways of AML cells. **a** Summary of metabolic enzymes and pathways studied after WIN-55 treatment. **b** Enzyme activity of glycolytic enzymes after 6 h incubation with 100 µM of WIN-55 alone or in combination with Olaparib pre-incubation treatment. Asterisk shows those cases in which this increase or decrease in activity caused by the cannabinoid was significantly reversed after preincubation with 10 µM Olaparib. Data are provided as mean ± SD of n = 4. **c** Extracelullar acidification rate (ECAR) values were measured during sequential injection of glucose and 2-deoxyglucose (2-DG) in U937 cells after vehicle, WIN-55 and/or Olaparib treatments. Data are mean ± SD for n = 4. **d** Enzymatic activity of G6PDH after 6 h incubation with 100 µM of WIN-55, 10 µM of Olaparib or the combination of both. Data are provided as mean ± SD of n = 3. Statistically significant differences were determined by Student’s *t* tests: *p ≤ 0.05, **p ≤ 0.005 and ***p ≤ 0.0005.

We next investigated if WIN-55 treatment also alters glycolysis, pentose phosphate pathway (PPP), and Krebs cycle (pathways summary showed in Fig. 6a). Moreover, considering that the activity of some of the enzymes involved in glycolysis can be regulated by PARylation, we explored the effect of WIN-55 alone or in combination with Olaparib.

To examine the effect of WIN-55 on glycolysis, a comprehensive study of the activity of enzymes involved in glycolysis upon 100 µM WIN-55 and/or 10 µM Olaparib treatment was performed (U937 cell line was used because studies in untreated cells were more robust in enzymes analysis). Overall activity of most glycolytic enzymes was altered upon WIN-55 exposure with the exception of enolase. Some of the enzymes including hexokinase (HK), glucose-6-phosphate isomerase (PGI), fosfofructoquinasa-1 (PFK), aldolase, triosephosphate isomerase (TPI), glyceraldehyde 3-phosphate dehydrogenase (GAPDH) and phosphoglycerate mutase (PGM) were significantly decreased whereas phosphoglycerate kinase (PGK) and pyruvate kinase (PK) were increased (Fig. 6b). Moreover, we also determined the reversion of the effect of WIN-55 on enzyme activities upon Olaparib pre-incubation (Fig. 6b).

To further investigate the effect of WIN-55 on glycolysis, we next determined the ECAR of AML cells upon WIN-55 and/or Olaparib treatments. Results showed that ECAR values were decreased in U937 cells upon WIN-55 treatment (Fig. 6c) and HL60 cells (Supplementary 6A) whereas no changes were observed in normal CD34^+^ cells (Supplementary Fig. 6B). Moreover, alteration of ECAR by WIN-55 was reverted upon Olaparib pre-incubation. All together, these results demonstrate that WIN-55 alters the glycolysis in AML cells and that these changes are reversed upon Olaparib pre-incubation, suggesting that PARP might play an important role in this effect.

Even though glycolysis is the central energy-producing pathway, cancer cells also rely on PPP, which provides an alternative pathway for glucose metabolism. Figure 6d shows how the activity of G6PDH decreases upon WIN-55 exposure in U937 cells and that this effect is reversed by Olaparib pre-incubation.

Next, we evaluated the effect of WIN-55 on the Krebs cycle. To do so, we supplemented the culture medium with different Krebs cycle metabolites (e.g. methyl pyruvate [MP], oxaloacetate acid [OAA] and dimethylsuccinate [DMS]) during 24 h before vehicle/WIN-55 treatment. The addition of these metabolites did not modify the effect of WIN-55 on U937 cells viability (Supplementary Fig. 6C), indicating that WIN-55 does not hinder the Krebs cycle by decreasing the availability of some of its metabolites.

### WIN-55 promotes cell death through PARP1-dependent cell death (Parthanatos)

Parthanatos is characterised by an irreversible loss of mitochondrial membrane potential, cellular NAD^+^ depletion, DNA fragmentation, increased PAR, AIF parylation and translocation of AIF from the mitochondrion to the nucleus.

**Fig. 7:**
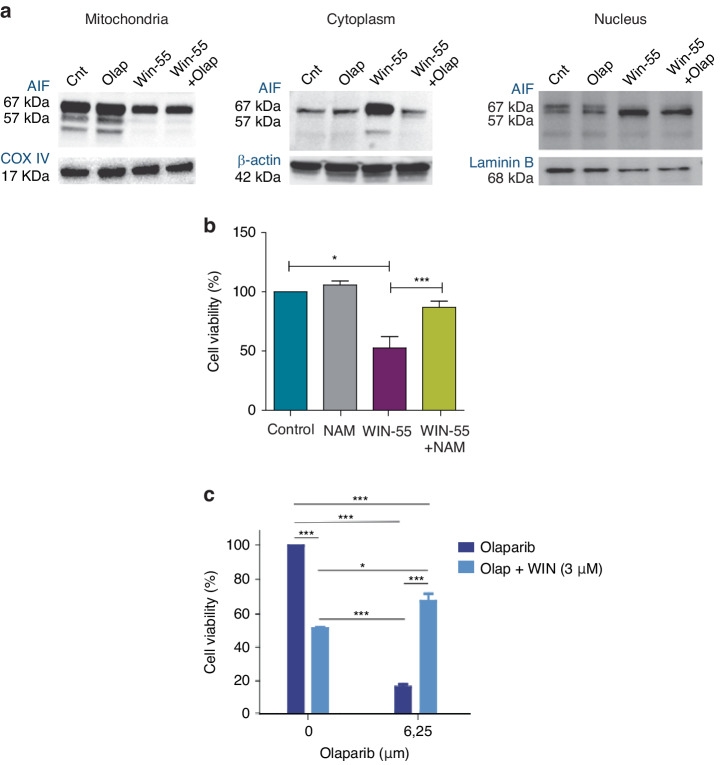
Parthanatos is involved in cytotoxic effect of WIN-55. **a** Western blotting analysis of AIF total protein fraction. HL60 cells were incubated 6 h with 10 µM Olaparib, 50 µM WIN-55 or both for the indicated time period (n = 3). **b** Cell viability measured by Cell Counting Kit-8 after incubation of nicotinamide (NAM) alone or in combination with WIN-55 for 18 h. Cell viability was assessed for U937 cells after 18 h incubation with nicotinamide (NAM) alone or in combination with 100 µM of WIN-55 treatment. Data are provided as mean ± SD of n = 3. Statistically significant differences were determined by Student’s *t* tests: *p < 0.05 and ***p ≤ 0.0005. **c** Cell viability measured by Cell Counting Kit-8 for eHAP cells after 48 h of incubation with Olaparib alone or in combination with 3 and 5 µM treatment with WIN-55. Data are given as mean ± SD of n = 3.

Considering the previously mentioned results, where we confirmed a loss of mitochondrial membrane potential upon WIN-55 treatment, as well as the inhibition of the effects of WIN-55 upon co-culture with Olaparib, we next investigated whether WIN-55 treatment triggers AIF-dependent apoptosis. For this purpose, we studied the expression of AIF in the nucleus, mitochondria, and cytoplasm after the sub-fractionation of AML cells upon 6 h of WIN-55 exposure. The results showed a decrease in protein levels of AIF in the mitochondria and an increase in the cytoplasmic and nuclear fraction (Fig. 7a), indicating that AML cells are suffering AIF-dependent apoptosis upon WIN-55 treatment.

Next, we analysed cellular NAD^+^ depletion, another hallmark of Parthanatos occurrence. For this purpose, WIN-55 treated cells were supplemented with nicotinamide (NAM), a precursor of NAD^+^; remarkably, the decreased viability after exposure to WIN-55 was recovered upon coculture with NAM (Fig. 7b), indicating that NAM prevents cannabinoid-induced cell death in AML cells.

Finally, to further confirm that WIN-55 activates PARP, we analysed whether or not WIN-55 was able to inhibit Olaparib-induced cell death which is related to PARP inhibition. For this purpose, we used the eHAP cell line, which is highly sensitive to PARP inhibition. Remarkably, preincubation with WIN-55 significantly decreased Olaparib-induced cell death. (Fig. 7c).

All these results highlight the fact that WIN-55 treatment produces PARP1-dependent cell death apoptosis.

## Discussion

In this study, we have demonstrated a powerful antileukemic effect of the cannabinoid WIN-55, exerted through its interaction with the CB receptor.

It has already been described that the CB2 receptor is highly expressed in AML cells [25, 26] which contrasts with the low expression in normal HSCs [11, 27]. This finding might, at least in part, explain the highly selective pro-apoptotic effect of WIN-55, since the viability of normal cells, including HSCs, was not affected after exposure to cannabinoids.

This antileukemic effect was also confirmed in vivo. For this purpose, we treated mice, xenografted with the HL60 cell line, once bone marrow infiltration was confirmed. In this model, WIN-55 treatment significantly prolonged survival compared to the vehicle-treated control and ARA-C-treated groups. Regarding the effect on normal HSC, no significant effects were observed neither on their viability nor on peripheral blood cell counts.

Regarding the mechanisms involved in this antileukemic effect, the transcriptomic study carried out identified differences in genes belonging to pathways that were grouped into: 1.Genes related with cell proliferation, apoptosis and damage in DNA, 2.Genes related to the correct functioning of organelles such as ER and mitochondria and 3.Genes involved in metabolic pathways.

As far as the first group is concerned, we confirmed that the PI3K/AKT pathway, MAPK and caspase activation were affected upon exposure to WIN-55.

On the other hand, the studies carried out to verify whether WIN-55 could cause DNA damage through detection of p-H2AX and PAR were not entirely conclusive. Interestingly, it has have described that WIN-55 induce apoptosis by triggering ROS-dependent DNA damage [28]. If WIN-55 does exert its prop-apoptotic effect through DNA damage in AML, a synergy with the PARP inhibitor Olaparib should have been observed. Surprisingly, Olaparib reversed cell death caused by the cannabinoid, thus opening a new avenue of investigation in this work, indicating that WIN-55 could act through PARP-thanatos. Interestingly, it has been described that AML cells express higher levels of this protein as compared to healthy HSC [29], that Olaparib it was able to induce cell death in AML. Furthermore, several studies suggest that PARP expression is a prognostic factor in AML [30–32]. Therefore, if WIN-55 induces PARP-thanatos, leukaemic cells would be sensitive to PARP either by default (justifying the effect of Olaparib) or by excess (explaining the effect of WIN-55).

Regarding the second group of genes affected in the transcriptomic study, related to damage to organelles, a significant increase in the levels of proteins involved in ER and UPR stress such as p-PERK, p-IRE1 and CHOP was observed.

Also, the studies carried out in the mitochondria revealed a significant reduction in the mitochondrial membrane potential and an increase in the levels of ROS production, as we previously observed in MM [11]. In contrast, ROS levels remained unchanged in normal HSCs.

The protective role that PARP plays in the mitochondria is well known [24]. Therefore, we checked whether preincubation with Olaparib could affect the accumulation of ROS produced by WIN-55; however, no significant differences were observed, indicating that PARP is not acting through mitochondrial damage in response to cannabinoid treatment.

The last group of genes highly affected in transcriptomic studies were those involved in metabolism. Ceramide metabolism is emerging as a second messenger on growth, differentiation [33] or cell death [34, 35]. In the current study, we confirmed that WIN-55 does affect the metabolism of ceramide, but this is not the main mechanism triggering cell death.

Finally, we proceeded to study glycolysis, since it was one of the pathways with the most genes affected after treatment with WIN-55.

The “Warburg effect” implies an increased dependence of cancer cells on anaerobic glycolysis. Although this is true for most tumour types, the LSCs in AML are more dependent on mitochondrial respiration for survival. In fact, LSCs fail to use the glycolytic machinery when energy from the mitochondria is blocked [36].

Our results show that WIN-55 impairs glycolysis and this effect is reversed upon co-culture with Olaparib. Moreover, it does alter the activity of all glycolytic enzymes with the exception of enolase. Furthermore, preincubation with Olaparib reversed this effect in the case of PGI, PFK, GAPDH and PK, indicating that they could be targets of PARP. The participation of PARP1 in bioenergetic control has already been suggested by Berger et al., who was the first to hypothesise that PARP1 hyperactivation was responsible for NAD^+^/ATP depletion [37].

More recent studies have shown a key role of PARP1 in the control of metabolism [38]. Poly (ADPribosyl) action or PARylation is a post-translational modification that is involved in a wide range of physiological and pathophysiological processes. In this regard, several studies have previously reported the effect that PARP1 exerts on glycolysis enzymes such as HK [39, 40], PFK [40], GAPDH [40–42], PK [38] and enolase [42] and PARP14 in PGI [43] and PK [44]. Remarkably we observed that Olaparib reversed the effect of WIN-55 in several of these enzymes. By contrast, it did not modify the effect of WIN-55 in mitochondrial respiration, thus confirming that the prior is the main mechanism involved in the antagonistic effect of Olaparib on WIN-55-induced apoptosis.

Our data also demonstrate an important modulation of the activity of glucose 6-phosphate dehydrogenase, which decreased dramatically after treatment with WIN-55. G6PDH is a rate-limiting enzyme for PPP, and therefore G6PDH levels also determine the flux of PPP and the rate of NADPH generation [45].

It has been described that AML cells undergo apoptosis after treatment with 6-aminonicotinamide, a potent inhibitor of G6PDH, while normal HSC are not affected. The importance of PPP in AML is reinforced by the observation that PPP genes are upregulated in 61% of AML patients. Therefore, PPP and especially G6PDH could be a potential therapeutic targets [46].

Finally, we confirmed the presence of Parthanatos, a programmed cell death characterised by the overactivation of PARP1, which leads to the depletion of NAD^+^ and cellular ATP, the release of AIF from the mitochondria to the nucleus and AIF PARylation.

Under physiological conditions, AIF maintains mitochondrial structure and plays an essential role in oxidative phosphorylation. On the contrary, under pathological conditions, AIF is a key mediator in caspase-independent cell death, and more specifically in PARP1-dependent cell death [47]. The PAR polymer is toxic to cells. Therefore, PAR polymer signalling along with mitochondrial AIF is the key event initiating fatal crosstalk between the nucleus and mitochondria in Parthanatos [47].

Another event in Parthanatos as mentioned above is the depletion of cellular NAD^+^. Upon activation, PARP1 transforms nicotinamide adenine dinucleotide (NAD^+^) into long PAR polymers and transfers them to a variety of nuclear proteins, including transcriptional histones, DNA polymerases, topoisomerases, DNA ligase-2, and itself [48, 49]. NAD^+^ is a cofactor of glycolysis and the Krebs cycle, which presents ATP to most cellular processes [50]. The overactivation of PARP1 results in the use of NAD^+^ and, therefore, the depletion of NAD^+^ and ATP levels [47].

Our data indicate that WIN-55 induces the release of AIF from the mitochondria. Moreover, when the cells were supplemented with nicotinamide, a precursor of NAD^+^, the viability of the cells treated with WIN-55 was recovered.

In summary, the cannabinoid derivative WIN-55 exerts a potent and selective antileukemic effect which depends on its interaction with the CB2 receptor and is mainly mediated through Parthanatos. Remarkably, treatment with WIN-55 affects enzymes involved in glycolysis and pentose phosphate pathways, leads to the translocation of AIF to the nucleus and to depletion of NAD^+^. These effects are not observed in normal HSC. Further studies will be required to explore the pathways linking CB2 and PARP activity.

### Supplementary information

Supplemental material merged

## Data Availability

The code of the Microarrays’ data deposition in a public repository is GSE252193.
