# Supplementary material for: Cannabinoids induce cell death in leukaemic cells through Parthanatos and PARP-related metabolic disruptions
Source: Br J Cancer. 2024 Mar 9;130(9):1529–41. doi: 10.1038/s41416-024-02618-6 (PMC11058274; doi:10.1038/s41416-024-02618-6)
Supplement: Supplementary file 1 — Supplemental material merged [file 41416_2024_2618_MOESM1_ESM.pdf]

MATERIAL AND METHODS

Drug and treatments.

For *ex vivo* assays, human primary cells were obtained from AML patients' bone marrow (BM) (Supplementary Table 1). Hematopoietic stem cells (HSC or CD34$^+$) were isolated from leukapheresis samples, B (CD19$^+$) and T (CD3$^+$) lymphocytes from buffy coats by immunomagnetic separation in the AutoMACS Pro separator (Miltenyi Biotec, Bergisch Gladbach, Germany) or FACS analysis, following manufacturer's instructions.

WIN-55,212-2 mesylate (hereafter referred to as WIN-55) was purchased from Tocris Bioscience (Bristol, UK). Cytarabine (ARA-C) was provided by the Department of Pharmacy of the University Hospital Virgen del Rocío.

CB1 antagonist (LY320135) and CB2 antagonist (SR144528) were purchase from Sigma-Aldrich. Myriocin (ISP-1), inhibitor of the serin-palmitoyl-transferase (SPT) enzyme, and Fumonisin B1 (FB1), inhibitor of ceramide synthases were obtained from Enzo Life Sciences (Lausen, Switzerland). Ceramide inhibitors BAF-312, PF-543, SKI-II, FTY-720, Ponesimod, Ozanimod, JTE-013 and ABC294640 were purchased from Selleckchem.

Olaparib, a PARP inhibitor, was also obtained from Selleckchem. Z-VAD(OMe)-FMK (pan-caspase inhibitor) was obtained from Abcam.

CB receptor expression levels quantification

Total RNA was isolated by using the QIAamp RNA Blood Mini Kit (Qiagen) following manufacturer´s instructions. The cDNA was generated from 2 μg of total RNA by using SuperSript IV VILO System (Invitrogen). cDNA solution (diluted 1:10; 2 μL) was used as a template for real-time quantitative PCR (RT-qPCR). Gene products were quantified by qPCR with the Applied Biosystems 7500 FAST Real-Time PCR System. Values were normalized to the expression of the human ABL housekeeping gene. Each experiment was performed in triplicate. Sequence of ABL oligonucleotides and Taqman probes used are listed below:

Fw ABL (ENF1003): 5´TGG AGA TAA CAC TCT AAG CAT AAC TAA AGG T 3´
RV ABL (ENR1063): 5´GAT GTA GTT GCT TGG GAC CCA 3´
ABL Probe (ENP541): 5´CCC TTC AGC GGC CAG TAG CAT CTG A 3´

TaqMan probes CNR2 Hs00275635_m1 and CNR1 Hs00275634_m1 (TermoFisher Scientific) were used for CB2 and CB1 gene expression, respectively.

Microarrays

RNA was amplified and labeled using the GeneChip® WT PLUS Reagent Kit (Thermo Fisher Scientific, Inc.) Amplification was performed with 100 ng of total RNA input following procedures described in the WT PLUS Reagent Kit user manual. The amplified cDNA was quantified, fragmented, and labeled in preparation for hybridization to GeneChip® Clariom S Human Array (Thermo Fisher Scientific, Inc.) using 5.5 µg of single-stranded cDNA product and following protocols outlined in the user manual. Washing, staining (GeneChip® Fluidics Station 450, Thermo Fisher Scientific, Inc.), and scanning (GeneChip® Scanner 3000, Thermo Fisher Scientific, Inc.) were performed following protocols outlined in the user manual for cartridge arrays. Arrays were normalized with RMA method from oligo package and differential expression analysis was performed using limma package. Log2 fold changes were used as input for Gene Set Enrichment Analysis, performed through cluster Profiler package. All analyses were done in R.

The code of the data deposition in a public repository is GSE252193.

Quantification of ceramides

HL60 and U937 cell lines (20x10^6 cells/condition) were treated with WIN-55 (50 µM) and/or ceramide inhibitors during different periods of time and lipids were extracted with a chloroform/methanol 2:1 solution. The chloroformic extracts of culture cell lysates were treated with 20 µL of internal standards and dried under a steady stream of nitrogen at room temperature. Reconstituted samples were analyzed using an Agilent liquid chromatographic system (1200 Series) consisting of binary pump (G1312A), connected to a triple quadrupole API 2000 mass spectrometer (Applied Biosystems) using an electrospray ionization interface in positive ionization mode (ESI+). Ceramides were separated on a Zorbax Eclipse XDB-C1 column from Agilent. Precursor and product ions used for quantification and confirmation purposes, and operating conditions are summarized in Supplementary Table 3.

Western blotting (WB)

Protein cell lysates were done using RIPA buffer supplemented with protease inhibitors (Roche, Mannheim, Germany), phosphatases (Thermo Scientific, Pierce Biotechnology, Rocford, IL, USA) and DNases (Roche, Basel, Switzerland). Then, protein lysates were quantified by (Bradford Bio Rad, Hercules, CA) and separated by SDS/PAGE electrophoresis. Immunoblots were subjected to chemiluminescence detection in ChemiDoc™ Touch Imaging System (Bio-Rad) and image quantification/normalization was performed in Image Lab software (Bio-Rad).

Cytoplasmic, mitochondrial and nuclear localization of AIF were assessed by the use of a Thermo Scientific mitochondria isolation kit or Thermo Scientific protein subcellular fractionation kit accordingly to the manufacturer's instructions.

SUPLEMENTARY FIGURE LENGEDS AND TABLES

Supplementary Figure 1

A) Effect of WIN-55 on HL60 and U937 cell lines. Cell viability and/or proliferation was assessed for HL60 and U937 cells after 18, 48 or 72h of incubation with WIN-55 at 0-10 µM doses by using the CCK-8 assay. B) The dot plots correspond to a representative example of the viability analysis by flow cytometry in the HL60 cell line exposed to different doses of WIN-55 for 15 minutes and analysed at 18 hours. Control conditions, 50 µM and 200 µM WIN-55 are shown in this figure. Right graph shows the analysis of live cells (7AAD-/Annexin V-) after treatment with different doses of WIN-55 in the HL60 cell line for 15 min and analysed at 18 hours. In all cases, the mean viability values of the control samples were taken as 100%. Data are provided as mean±SD of n=4. Statistically significant differences were determined by Student's t-tests: ** $p < 0.005$ and *** $p \leq 0.0005$. C) Example of the identification of blasts from BM of patients with AML by FACS.

Supplementary Figure 2

A) Summary of the toxicity study performed in healthy mice. B) Analysis of PB cell counts in BALB/c mice control or exposed to 5 mg/kg/day WIN-55 for 7 or 28 days. In all the conditions where the statistical significance is not detailed, it is because the data were not significant. WBC (white blood cells), GRA (granulocytes), LYM (lymphocytes), MON (monocytes), HGB (hemoglobin), and PLT (platelets) were studied. C) Schema of surface markers for each HSC subpopulation in mouse. D) The plots show the percentage of cells from the different BM subpopulations after vehicle or WIN-55 treatment for 7 and 28 days. We analysed bone marrow LKS cell subpopulations (long-term (LT-HSC), short-term (ST-HSC) and multipotent progenitor (MPP) LKS populations), LK subpopulations (common myeloid progenitor (CMP)), megakaryocyte/erythroid progenitors (MEP) and granulocyte/macrophage progenitor population (GMP)) and common lymphoid progenitors (CLP) by flow cytometry. An increase of total $Lin^-Kit^+Sca\text{-}1^-$ (LK) cells was observed after 7 days of treatment, which corresponded with the growth in granulocyte-monocyte precursor (GMP) and common myeloid progenitor (CMP) subpopulations. In all the conditions where the statistical significance is not detailed, it is because the data were not significant. Data are provided as mean±SD of n=4. Statistically

significant differences were determined by Student's t-tests: * $p < 0.05$, ** $p < 0.005$ and *** $p \leq 0.0005$.

Supplementary Figure 3

Dysregulated pathways identified upon WIN-55 treatment in HL60 (AML) cell line. Data was analysed by Gene Set Enrichment Analysis (GSEA) using Gene Ontology (GO) database. Volcano plots show concordant differences between WIN-treated and Control samples for genes associated with biological processes such as response to ER stress (upregulation), metabolic process (downregulation), DNA repair (downregulation), mitochondrion organization (downregulation), or cell population proliferation (downregulation). Data are provided as mean±SD of n=3.

Supplementary Figure 4

A) HL60 cells were treated or not with 5 µM WIN-55 in combination of 6,25 µM of Olaparib, Talazoparib and Niraparib. B) CD34+ cells from apheresis were treated with 50 µM WIN-55 for 15 or 30 minutes and then stained with 5 µM MitoSOX probe to detect mitochondrial superoxide. Data are mean±SD for n=3. C) Oxygen consumption rate (OCR) values were measured in HL60 cells during sequential injection of oligomycin, CCCP, and Rot+Ama in U937 cells after WIN-55 and/or Olaparib treatments using a Seahorse Analyzer. Basal and maximal respiration of cells were calculated. D) Basal oxygen consumption rate (OCR) values were measured in sorted $CD34^+$ after WIN-55 and/or Olaparib treatments using a Seahorse Analyzer. Data are provided as mean±SD of n=3. Statistically significant differences were determined by Student's t-tests: * $p < 0.05$ and ** $p \leq 0.005$.

Supplementary Figure 5

A) Quantification of the levels of different types of ceramides by HPLC / MS-MS in the U937 cell line after incubation with 50 µM of WIN-55 at the times indicated. Data are mean±SD for n=3. B) The graphs show the levels of S1P and C) C1P in the U937 line quantified by HPLC/MS-MS after treatment for 6 and 18 hours with 50 µM WIN-55. Data are mean±SD for n=3. D) Number of colonies grown from a single cell treated with WIN-55 in combination with vehicle, myriocin, or FB1. Data are mean±SD for n=4. Only the cases in which the statistical study was significant are indicated. Statistically significant differences were determined by Student's t-tests: * $p < 0.05$ and ** $p \leq 0.005$.

Supplementary Figure 6

A and B) Extracellular acidification rate (ECAR) values were measured during sequential injection of glucose and 2-deoxyglucose (2-DG) in HL60 cells (A) and sorted $CD34^{+}$ cells (B) after vehicle, WIN-55 and/or Olaparib treatments. Data are mean±SD for n=4. C) Viability studies after supplementation of the Krebs cycle metabolites methyl pyruvate (MP), dimethyl succinate (DMS) and oxaloacetate acid (OAA), all at a concentration of 5 mM, alone or in combination with 10 µM WIN- 55 or vehicle in the cell line U937 analysed by labelling with 7AAD/Annexin by flow cytometry. Data are mean±SD for n=3.

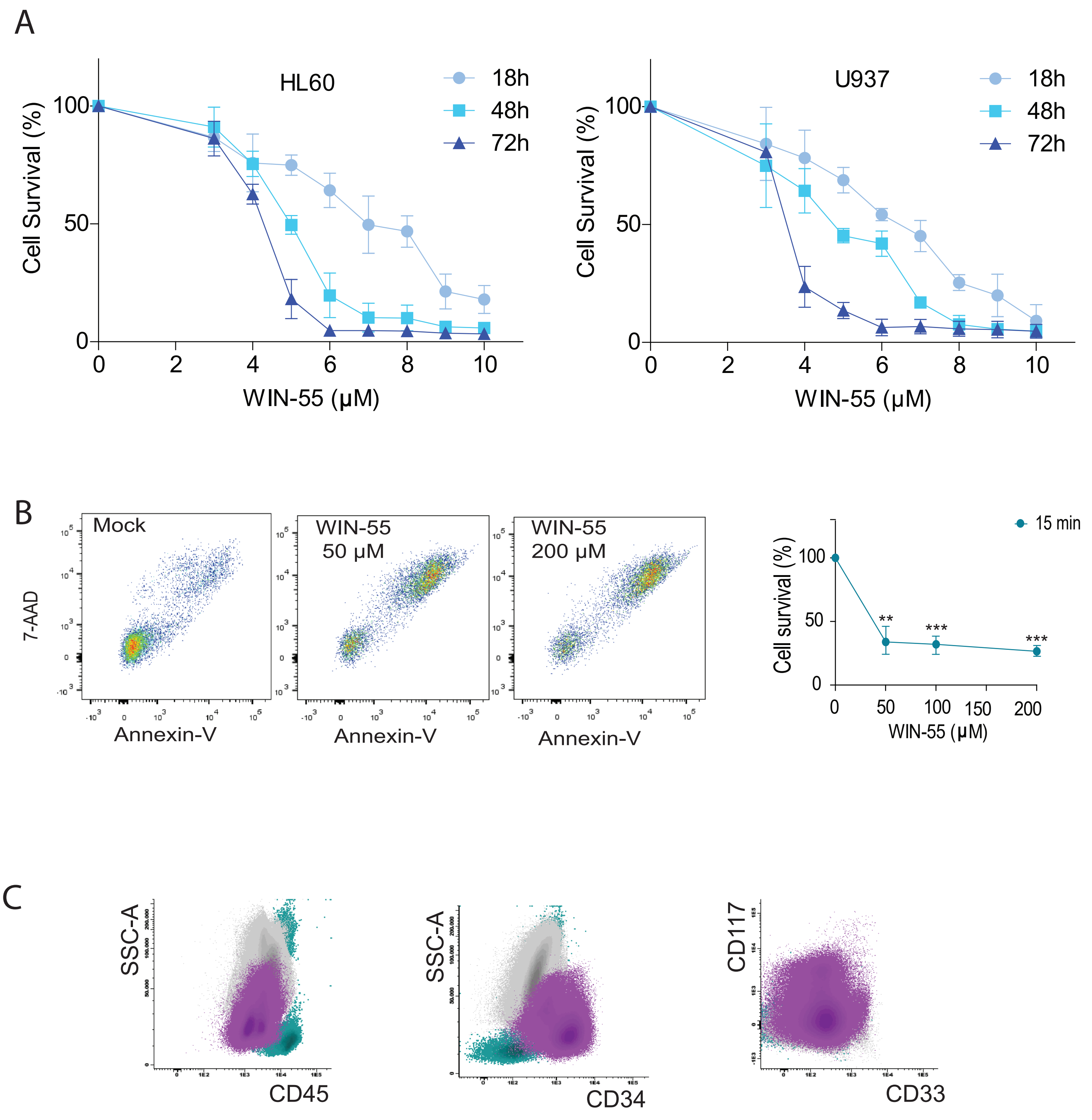
A
HL60
U937
18h
48h
72h
Cell Survival (%)
WIN-55 (μM)
B
Mock
WIN-55 50 μM
WIN-55 200 μM
7-AAD
Annexin-V
15 min
Cell survival (%)
WIN-55 (μM)
**
***
***
C
SSC-A
CD45
CD34
CD117
CD33

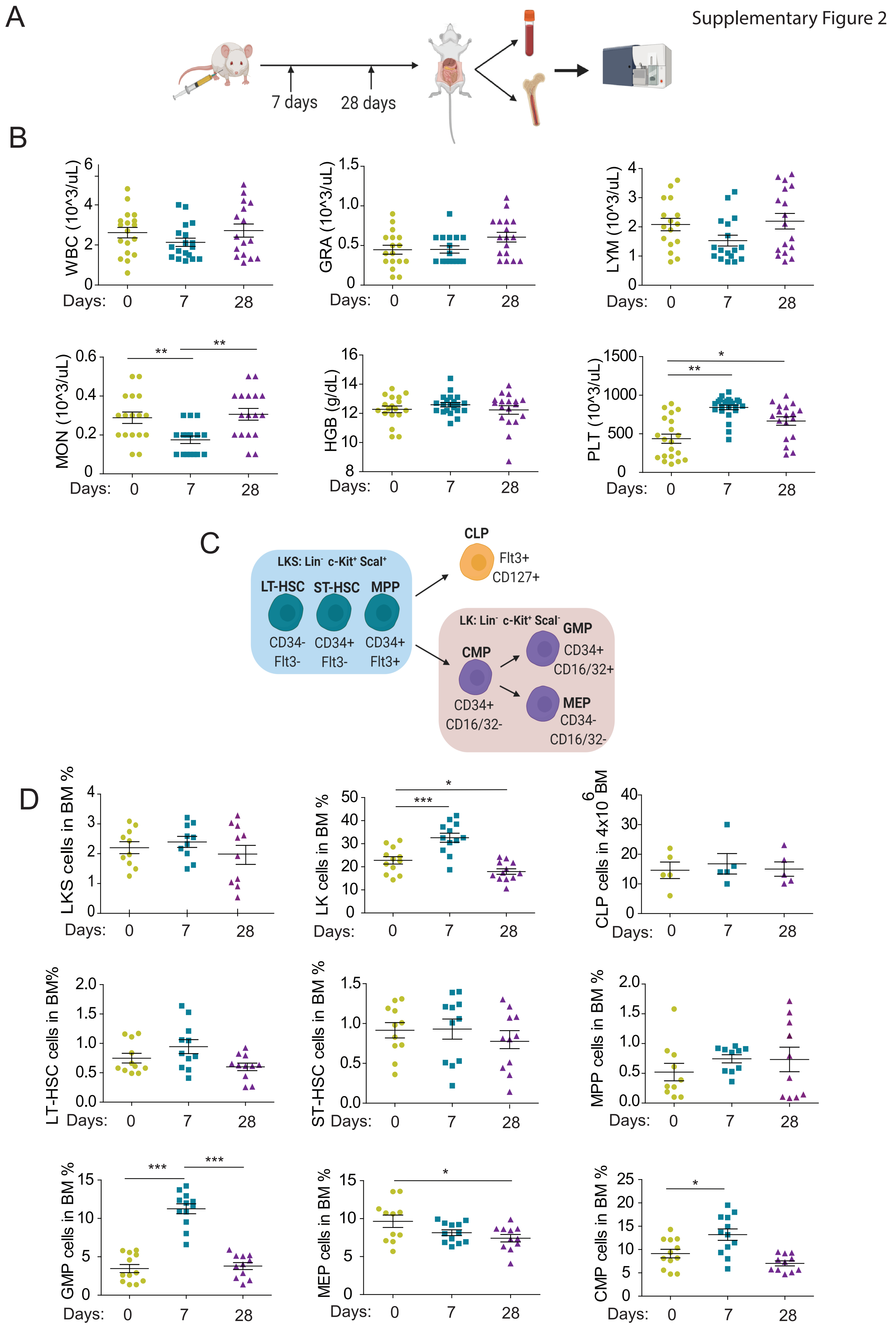
Supplementary Figure 2
A
7 days
28 days
B
WBC (10^3/uL)
GRA (10^3/uL)
LYM (10^3/uL)
MON (10^3/uL)
HGB (g/dL)
PLT (10^3/uL)
Days: 0 7 28
C
LKS: Lin- c-Kit+ ScaI+
LT-HSC
ST-HSC
MPP
CD34- Flt3-
CD34+ Flt3-
CD34+ Flt3+
CLP
Flt3+ CD127+
LK: Lin- c-Kit+ ScaI-
CMP
CD34+ CD16/32-
GMP
CD34+ CD16/32+
MEP
CD34- CD16/32-
D
LKS cells in BM %
LK cells in BM %
CLP cells in 4x10^6 BM
LT-HSC cells in BM%
ST-HSC cells in BM %
MPP cells in BM %
GMP cells in BM %
MEP cells in BM %
CMP cells in BM %

Supplementary Figure 3

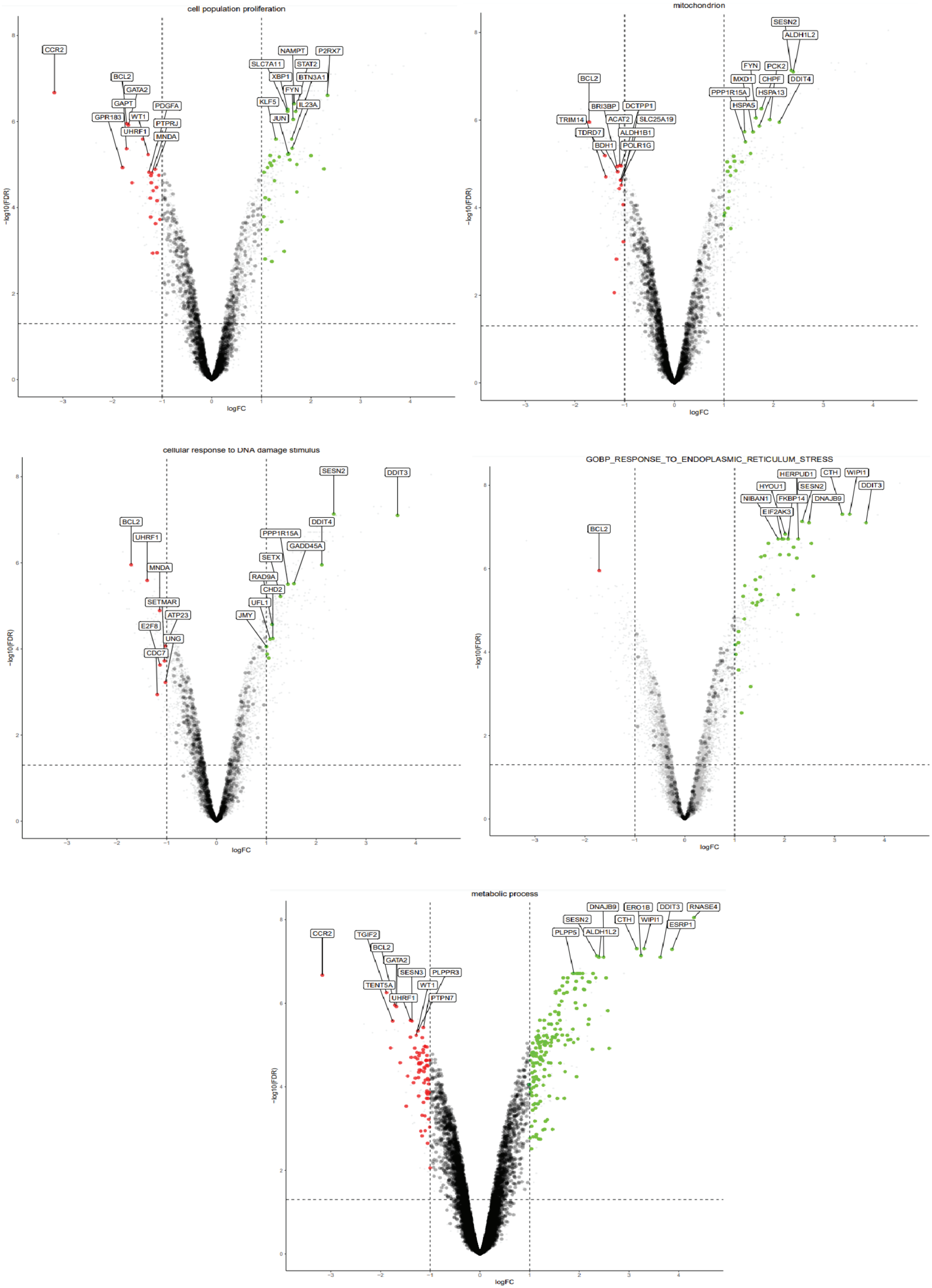

Supplementary Figure 4

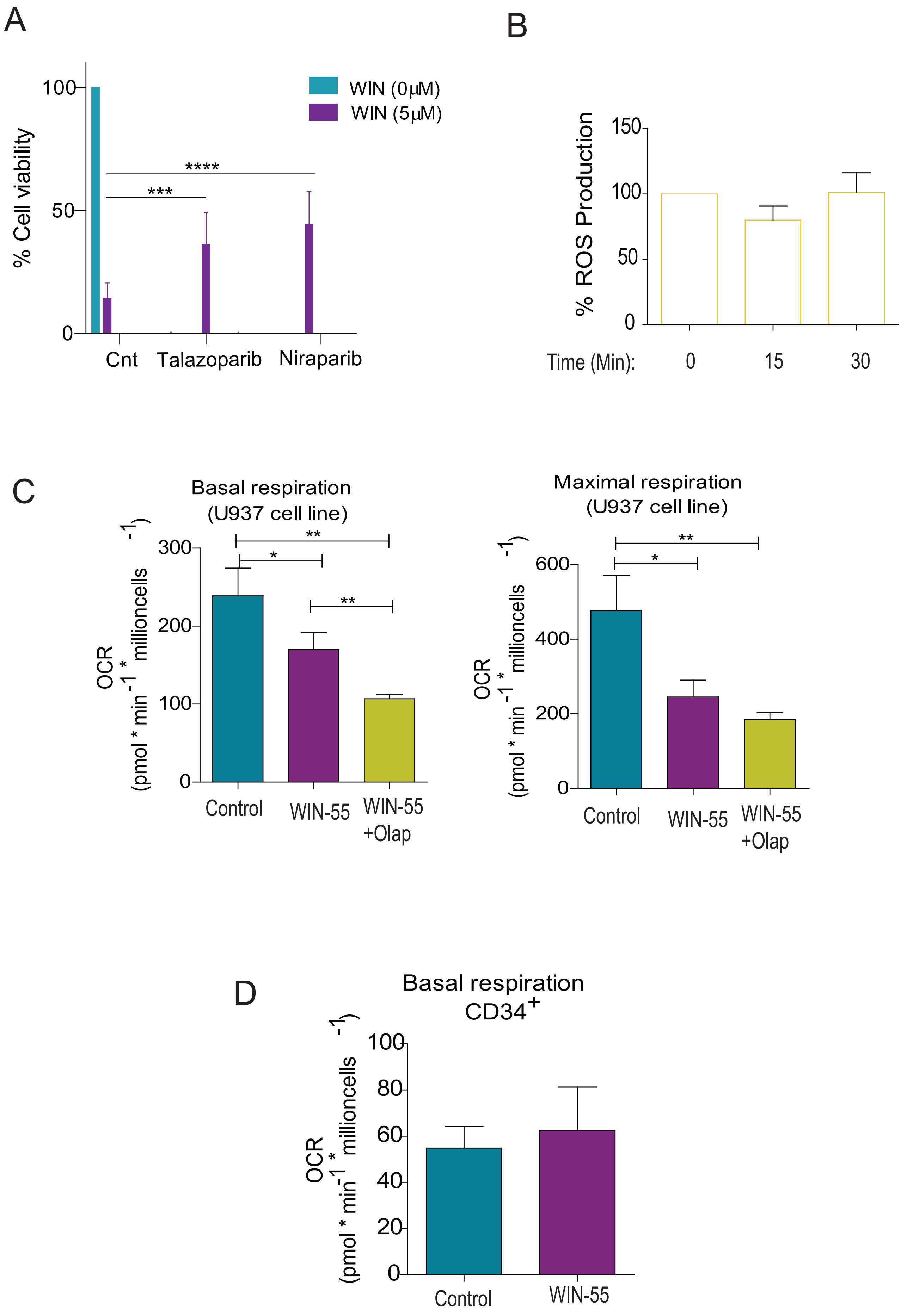

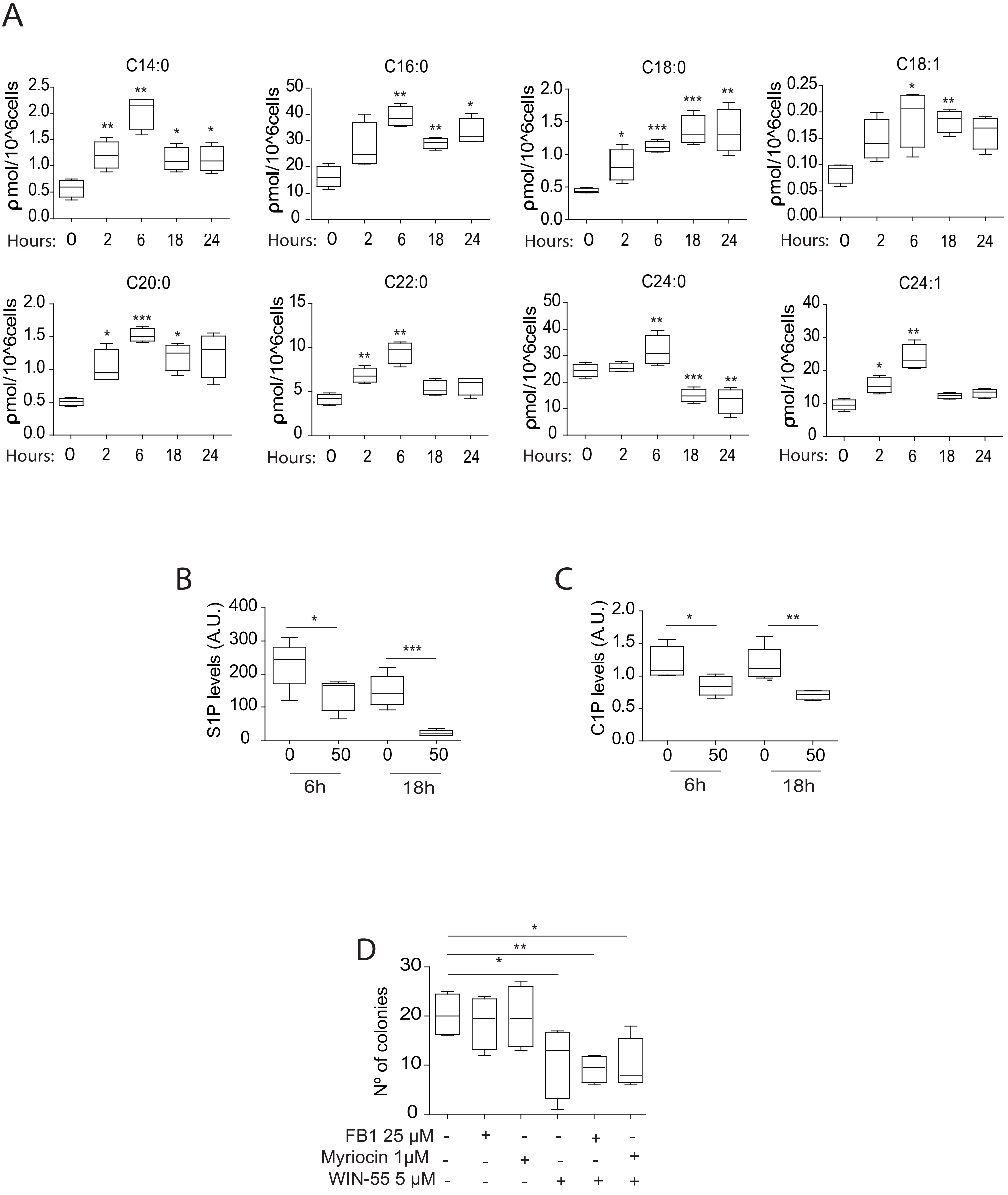
A
C14:0
C16:0
C18:0
C18:1
C20:0
C22:0
C24:0
C24:1
ρmol/10^6cells
Hours: 0 2 6 18 24
B
S1P levels (A.U.)
0 50 0 50
6h
18h
C
C1P levels (A.U.)
0 50 0 50
6h
18h
D
Nº of colonies
FB1 25 μM - + - - + -
Myriocin 1μM - - + - - +
WIN-55 5 μM - - - + + +

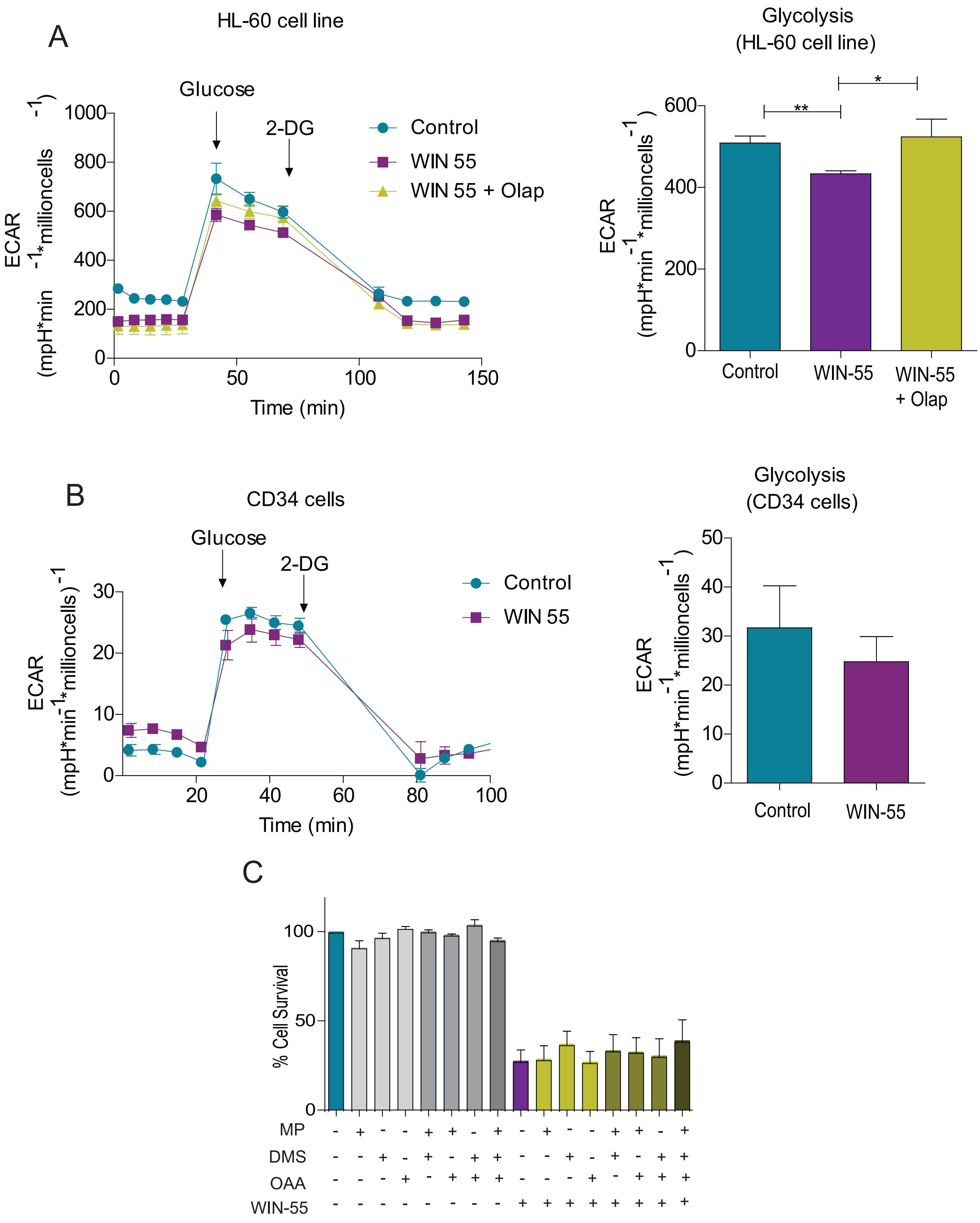
A
HL-60 cell line
Glucose
2-DG
Control
WIN 55
WIN 55 + Olap
ECAR (mpH*min-1*millioncells-1)
Time (min)
Glycolysis (HL-60 cell line)
Control
WIN-55
WIN-55 + Olap
B
CD34 cells
Glycolysis (CD34 cells)
C
% Cell Survival
MP
DMS
OAA
WIN-55

Supplementary table 1. Clinical characteristics of patients from which primary AML cells were obtained.

UPN: unique patient number; del=deletion, t=translocation, amp=amplification.

PATIENT ID	Cariotype/Molecular Assays	Gene Mutations	% BM Infiltration	% Cell Viability 50 µM WIN-55
UPN-1	45,X,-Y,t(8;21)(q22;q22)[18]/46,XY[2]	N.P.M.F	57,40	47,60
UPN-2	46,XX,del(7)(q22q32)[13]	GATA2, PTPN11	40,40	54,50
UPN-3	46,XY,t(2;8;21;8)(q31;q23;q22;q22)[20]	N.D	52,20	28,43
UPN-4	PML/RARA-t(15;17)(q22;q21)	N.P.M.F	93,60	25,07
UPN-5	46, XY	NPM1, TET2, TP53	64,80	8,42
UPN-6	46,XY,inv(16)(p13q22)[12]	FLT3, KIT, KRAS	41,40	75,96
UPN-7	46,XX[20]	ASXL1, DNMT3A, KRAS, NPM1	71,00	93,00
UPN-8	46,XY,t(8;21)(q22;q22)[9]/46,XY[1]	CEBPA, TET2	78,80	2,21
UPN-9	46,XX,ins(10;11)(q23;p12p12)[20]	N.P.M.F	89,90	2,59
UPN-10	46,XX[10]	DNMT3A, NPM1, PTPN11	33,90	13,19
UPN-11	46,XX[20]	NPM1, WT1, TET2	81,00	11,22
UPN-12	45,XY,del(5)(q15q33),dic(17;20)(p13;q13.2)[19]/46,XY[1]	N.D	19,80	36,51
UPN-13	46,XX[15]	NPM1, GATA2, TET2	81,00	52,19
UPN-14	N.D	RUNX1, IDH1, DNMT3A	88,00	8,38
UPN-15	46,XY,-7[10]	RUNX1, FLT3-TKD, NRAS, PTPN11	74,00	0,00
UPN-16	46,XX[3]	DNMT3, TET2	81,40	69,42
UPN-17	N.D	N.D	96,90	25,30
UPN-18	47,XY,+8[12]/48,XY,+6,+8[8]	N.P.M.F	95,20	13,79
UPN-19	N.D	FLT3, NPM1, TET2, ETV6, CALR	96,10	46,01
UPN-20	46, XY	IDH1	66,90	28,09
UPN-21	46,XX,t(6;11)(q27;q23)[20]	N.P.M.F	90,00	98,95
UPN-22	N.D	IDH2, RUNX1	64,30	24,21
UPN-23	46, XX	NPM1, IDH1	58,20	66,70
UPN-24	46, XY	SRSF2, IDH2	95,00	76,79
UPN-25	46,XY[20]	SRSF2, IDH2, CEBPA, STAG2	83,1	30,16
UPN-26	46,XX[15]	NPM1, FLT3-ITD, TET2	94,30	93,54
UPN-27	t(8,21) AML1-ETO (3140000 copies/ABL:1360000)	KIT, NF1	23,30	10,16
UPN-28	46,XY[16]	NPM1, FLT3-ITD, IDH2	89,20	6,43
UPN-29	46,XY[20]	NPM1, FLT3-ITD, STAG2	84,30	11,27
UPN-30	N.D	NRAS, DNMT3A, FLT3-ITD, NPM1	43,50	23,75

UPN-31	46,XX,add(7)(q22),add(12)(p13),-17,+mar,20-60dmin[14]	TP53, DNMT3A	32,80	50,96
UPN-32	46,XX[20]	CALR	19,00	48,87
UPN-33	N.D	FLT3-ITD, NPM1, TET2, WT1	79,00	26,99
UPN-34	46,XY,+Y,der(15;22)(q10;q10)[20]	FLT3-ITD, NPM1, DNMT3A	54,20	48,67
UPN-35	N.D	NPM1, IDH1, GATA2	79,00	6,58
UPN-36	47,XY,+8[2]/46,XY[18]	NPM1, FLT3-TKD2, WT1	91,75	43,20
UPN-37	N.D	RUNX1, CBL, STAG2, TET2	31,25	84,67
UPN-38	46,XX[15]	CEBPA	76,10	12,13
UPN-39	46,XX,der(3)inv(3)(q21.3q26.2)t(3;14)(q26.2;q31),t(3;14)(q26.2;q31)[15]	KRAS, IKZF1	54,10	45,14
UPN-40	46,XX[20]	CEBPA, CSF3R, NRAS, WT1,	55,00	66,60

Supplementary table 2. List of antibodies used in the detection of hematopoietic populations in mice.

Antibody	Fluorophore	Company	Catalog number
**7AAD**	PerCP Cy5	BD Biosciences	51-68981E
**Annexin V**	PE	BD Pharmingen	556422
**B220**	FITC	Immunostep	M45RF-05MG
**B220**	PE	BD Pharmingen	553089
**CD11b**	FITC	Immunostep	M11BF-05MG
**CD11b**	PE	Immunostep	M11BPE-02MG
**CD11c**	FITC	Immunostep	M11CF-05MG
**CD127**	BV510	BD Biosciences	563353
**CD16/32**	V450	BD Biosciences	560539
**CD3**	FITC	eBioscience	11-0031-82
**CD3**	PerCP Cy5	BD Pharmingen	551163
**CD34**	APC	eBioscience	50-0341-82
**CD4**	FITC	Immunostep	M4F-05MG
**CD4**	APC	BD Biosciences	553051
**CD45**	APC	BD Biosciences	559864
**CD8**	FITC	BD Biosciences	553031
**CD8**	PE	Immunostep	M8APE-02MG
**C-Kit**	PerCP Cy5	BioLegend	105824
**FLT3**	PE	Immunostep	1399990072
**GR1**	FITC	Immunostep	MLY6G6CF-05MG
**IgM**	APC	BD Biosciences	550676
**ScaI**	PE Cy7	BD Biosciences	558162
**Ter119**	FITC	Immunostep	MECF-05MG

Supplementary table 3. Optimization of MRM transitions for detection of ceramides. (DP: declustering potential; FP: focusing potential; EP: entrance potential; CE: collision energy; CXP: cell exit potential; IS: Internal Standard).

Ceramide	Monitored transition	DP	FP	EP	CE	CXP
14:0	492.5 → 264.3	86	370	8	31	6
	492.5 → 82.2	86	370	8	69	2
	492.5 → 56.1	86	370	8	69	6
16:0	520.5 → 264.2	121	370	9	33	6
	520.5 → 82.2	121	370	9	69	2
	520.5 → 55.0	121	370	9	95	6
18:0	548.6 → 264.4	86	370	9	37	6
	548.6 → 82.0	86	370	9	83	8
	548.6 → 55.0	86	370	9	97	6
18:1	546.5 → 264.3	106	370	8.5	37	6
	546.5 → 82.2	106	370	8.5	75	2
	546.5 → 55.1	106	370	8.5	101	6
20:0	225.2 → 55.1	31	360	10	53	6
	225.2 → 61.1	31	360	10	35	8
	225.2 → 100.2	31	360	10	23	4
22:0	604.5 → 264.3	136	370	12	35	6
	604.5 → 82.2	136	370	12	87	2
	604.5 → 55.1	136	370	12	111	6
24:0	632.5 → 264.3	146	250	11	37	6
	632.5 → 82.2	146	250	11	91	2

	632.5 → 55.1	146	250	11	109	6
24:1	630.5 → 264.2	136	370	9.5	45	8
	630.5 → 82.2	136	370	9.5	75	0
	630.5 → 67.0	136	370	9.5	123	0
17:0 (IS)	534.4 → 264.3	101	370	10	37	6
	534.4 → 82.2	101	370	10	77	2
	534.4 → 55.1	101	370	10	103	6
